# Phage Treatment Trial to Eradicate LA-MRSA from Healthy Carrier Pigs

**DOI:** 10.3390/v13101888

**Published:** 2021-09-22

**Authors:** Henni Tuomala, Marie Verkola, Anna Meller, Jasper Van der Auwera, Sheetal Patpatia, Asko Järvinen, Mikael Skurnik, Annamari Heikinheimo, Saija Kiljunen

**Affiliations:** 1 Division of Clinical Microbiology, HUSLAB, Helsinki University Hospital, Haartmaninkatu 3, 00290 Helsinki, Finland; henni.tuomala@helsinki.fi (H.T.); mikael.skurnik@helsinki.fi (M.S.); 2 Human Microbiome Research Program, Faculty of Medicine, University of Helsinki, P.O. Box 21 (Haartmaninkatu 3), 00014 Helsinki, Finland; jasper.vanderauwera@hotmail.com (J.V.d.A.); sheetal.patpatia@helsinki.fi (S.P.); 3 Department of Food Hygiene and Environmental Health, Faculty of Veterinary Medicine, University of Helsinki, P.O. Box 66 (Agnes Sjöbergin katu 2), 00014 Helsinki, Finland; marie.verkola@helsinki.fi (M.V.); annamari.heikinheimo@helsinki.fi (A.H.); 4 Laboratory Animal Center, University of Helsinki, 00014 Helsinki, Finland; anna.meller@helsinki.fi; 5 Department of Infectious Diseases, Inflammation Center, Helsinki University Central Hospital and University of Helsinki, Haartmaninkatu 4, 00029 Helsinki, Finland; asko.jarvinen@hus.fi; 6 Finnish Food Authority, Laboratory and Research Division, Microbiology Unit, P.O. Box 200, 00027 Helsinki, Finland

**Keywords:** LA-MRSA, phage, phage therapy, pig, antibiotic resistance

## Abstract

The increase of livestock-associated methicillin-resistant *Staphylococcus aureus* (LA-MRSA) causes a threat to human health. LA-MRSA can be transmitted from animals to animal caretakers, which may further spread MRSA to communities and health care facilities. The objective of this work was to study the efficacy of phage treatment in the eradication of LA-MRSA from healthy carrier pigs. A total of 19 MRSA -positive weanling pigs were assigned to a test (*n* = 10) and a control group (*n* = 9). A phage cocktail containing three *Staphylococcus* phages, or a control buffer was administered to the nares and skin of the pigs three times every two days, after which the phage and MRSA levels in nasal and skin swab samples were monitored for a three-week period. The sensitivity of the strains isolated during the follow-up period to the phage cocktail and each phage individually was analyzed and the pig sera were tested for antibodies against the phages used in the cocktail. The phage treatment did not cause any side effects to the pigs. Phages were found in the skin and nasal samples on the days following the phage applications, but there was no reduction in the MRSA levels in the sampled animals. Phage-resistant strains or phage-specific antibodies were not detected during the experiment. The MRSA load in these healthy carrier animals was only 10–100 CFU/swab or nasal sample, which was likely below the replication threshold of phages. The effectiveness of phage treatment to eradicate MRSA from the pigs could thus not be (reliably) determined.

## 1. Introduction

The global spread of antimicrobial resistance in bacteria jeopardizes modern healthcare as infections become more difficult and even impossible to treat. Many of these resistant bacteria are zoonotic, i.e., they transmit between humans and animals in both directions. Methicillin-resistant *Staphylococcus aureus* (MRSA) is a well-known antibiotic-resistant pathogen causing both community- and healthcare-associated infections and outbreaks in healthcare facilities. For the past 15 years, it has increasingly been found in livestock, predominantly in pigs, veal calves, poultry, and horses [1,2,3,4,5]. In Europe, these livestock-associated MRSA (LA-MRSA) strains, mainly belong to clonal complex (CC) 398, and pose a threat to humans especially those working in close contact with colonized animals [6,7,8,9]. The humans who get these zoonotic infections are mainly colonized asymptomatic carriers, but they may further spread the resistant pathogens into the community and into healthcare facilities [10,11]. In addition to livestock, LA-MRSA has been detected in meat such as pork and veal (ECDC, 2021). However, food-borne LA-MRSA infections are not probable [12,13,14].

The prevalence of LA-MRSA in pigs in Europe varies by country. Numbers reported by the European Food Safety Authority (EFSA) (2021) ranged from 0.1% in pig herds in Norway to 94.5% in breeding pig herds in Denmark in 2019. In Finland, the prevalence of LA-MRSA in fattening pig batches at slaughter was 77.0% in 2016–2017 [15]. The proportion of LA-MRSA of all human MRSA cases in Finland has been rising steadily and reached 6.8% in 2019 [16]. Considering the low incidence of MRSA in general, 1391 cases in 2019 [16], the increase in LA-MRSA cases is a reason for concern.

One potential solution for the eradication of LA-MRSA colonization from livestock might be phage therapy, the utilization of viruses that infect bacteria (bacteriophages or phages). The history of phage therapy is over 100 years long [17], and the interest in it is rising upon the emerging number of antibiotic-resistant pathogenic bacteria [18]. There are a number of studies where phage therapy has been used in farm animals. In 1983, Smith and Huggins published a study where phages were successfully used to treat *Escherichia coli*-induced diarrhea in calves, piglets, and lambs [19]. The largest scale animal experiments have often been performed with poultry. Two field trials, both including over 34,000 chickens, were conducted in Colombia to test the reduction of *Salmonella* by SalmoFREE, a phage cocktail composed of six phages [20]. The results of these trials were promising but did not produce definite conclusions due to simultaneous antibiotic treatments and putative phage contamination or carry-over. In a smaller experiment, two different phage products (a single phage and a four-phage cocktail) were used to treat *E. coli*-induced infection from 360 Japanese quails divided into six treatment groups [21]. The mortality of the quails was found to decrease in both phage-treated groups when compared to non-treated control birds, with the phage cocktail group showing a higher survival percentage than the monophage group.

*S. aureus*, especially MRSA, is one of the most intensively studied target bacteria for phage therapy [22]. There are several *S. aureus*–specific phages known, all of which belong to the order of *Caudovirales*. Most of the phages that infect *S. aureus* are temperate phages having the siphovirus morphotype. However, from a phage therapy point of view, the most interesting *S. aureus*-specific phages are the lytic, broad host range phages belonging to the family *Herelleviridae* (earlier classified as members of *Myoviridae* family) [23].

Many of the published studies concerning phage therapy of *S. aureus* infections in humans are individual case studies. For example, in a recently published series of case studies, five patients having different kinds of *S. aureus* infections related to cardiothoracic surgery were treated with personalized phage cocktails [24]. The *S. aureus* infection was considered healed for four out of the five patients, but one patient died from *S. aureus* sepsis 1.5 months after the treatment, despite the initial reduction of *S. aureus* in the infection loci. The relatively broad host range of some *S. aureus* phages make *S. aureus* also an interesting target for double-blinded clinical studies. There are currently five clinical trials concerning phage therapy of *S. aureus* infections, either alone or in combination with other bacterial pathogens, in the ClinicalTrials database (ClinicalTrials.gov Identifiers NCT04815798, NCT00663091, NCT04803708, NCT02664740, and NCT04636554). However, these studies are still in their early phases and there are no reported results available at the time of writing.

There are a few studies concerning phage therapy of *S. aureus* on production animals, but the results have been somewhat contradictory. Phage K was shown to cure subclinical *S. aureus* mastitis of 16.7% of lactating cows (*n* = 18), compared to 0% recovery in the control group (*n* = 20). However, the result was not statistically significant. [25] Drilling et al. published two studies about the use of *S. aureus*-specific phage cocktails in sheep. One of the articles was a safety study showing that the phage cocktail did not cause any inflammatory infiltration or tissue damage when applied to sinuses of healthy sheep, and the other study showed that phage treatment reduced the number of subepithelial acute inflammatory cells and biofilm production caused by *S. aureus* in sheep sinusitis [26,27]. A conflicting result was obtained in an MRSA sinusitis model in piglets, where phage treatment did not reduce bacterial counts even though the phage cocktail efficiently killed the MRSA strain in vitro [28]. These examples clearly show that more studies are needed in order to understand the efficacy of phage treatments in production animals.

The aim of this work was to evaluate if phage treatment could be utilized to eradicate LA-MRSA skin and/or nasal colonization from healthy carrier pigs. To our knowledge, there are no previous studies where phages would have been studied to eradicate the carriage of MRSA. As the porcine immune system has over 80% similarity to humans, pigs are considered good models in immunologic research [29]. We thus anticipated that the work would add to the understanding of human phage therapy as well. The study was carried out with animals coming from a farm having earlier identified LA-MRSA-positive status and is a continuation of our previous work showing that LA-MRSA can persist in the farm environment with low bacterial quantities [30].

## 2. Materials and Methods

### 2.1. Phages and Host Bacteria Used in the Work

Phage vB_SauM_fRuSau02 (fRuSau02) was isolated from a commercial phage therapy product [31] and phage vB_SauP_EBHT (EBHT) (GenBank accession MT926124) was obtained from the collection of DSMZ. Clinical *S. aureus* strain 13KP [31] and MRSA 19A2, isolated from a healthy pig, [30] were used as host strains for fRuSau02 and EBHT, respectively. Phage EBHT was recently shown to change its host range depending on which host it is produced (Tuomala et al., manuscript under preparation), and the phage cultured in strain 19A2 is denoted as mEBHT. Phage фYeO3-12 [32,33] cultured in *Yersinia enterocolitica* strain 6471/76-c [34] was used to optimize an in-house enzyme-linked immunosorbent assay (ELISA) for antibody detection.

Phages vB_SauS_fPfSau02 (fPfSau02), vB_SauS_fPfSau03 (fPfSau03), and vB_SauS_fPfSau04 (fPfSau04) were isolated from pig feces collected from the same farm from which the MRSA strains originate with standard methods [35]. The isolation hosts for fPfSau02, fPfSau03, and fPfSau04 were MRSA strains 18B2, 19A2, and 5A1 [30], respectively. Strain 19A2 was later used as the standard host for phage fPfSau02.

### 2.2. Phage Titration and Host Range Analysis

The phage titer was determined with the standard double layer method in Lysogeny broth (LB) as described in [35]. LB agar plates were prepared by supplementing LB with 1.5% (*w*/*v*) agar and soft agar by supplementing LB with 0.4% (*w*/*v*) agar. All incubations were conducted at 37 °C. The phage host range was determined either with spot assay [35] using a double-layer method and 1:100–diluted phage lysate/cocktail or with a liquid culture method for strains that did not grow evenly in soft agar. The liquid culture assay was performed in Brain Heart Infusion Broth (BHI) as described elsewhere [31]. Phage infection was considered clear if the phage produced a clear spot in spot assay or >70% inhibition in bacterial growth in liquid culture. The infection was considered weak if the phage produced turbid spot or 40–60% growth infection in spot assay and liquid culture, respectively. The strain was considered resistant, if there was no obvious spot or if the growth inhibition in liquid culture was less than 20%.

### 2.3. Phage Genome Sequence Analysis

Phage DNA was isolated manually from fresh phage lysates by standard phenol-chloroform extraction and ethanol precipitation method [35], after which the residual phenol and chloroform were washed away with Vivacon 500 DNA concentrator (Sartorius). Next-generation sequencing was performed at Eurofins GATC Biotech (Constance, Germany). De novo genome assembly was carried out using the A5 (Andrew And Aaron’s Awesome Assembly) pipeline [36] and the genome termini were determined with PhageTerm [37]. The assembly was verified by mapping the original reads back to the assembled genome by Geneious 10.1.3 assembler. The genome was annotated using RASTtk [38] and BLASTP [39]. The presence of genes encoding virulence factors in the fPfSau02 genome was searched with the VFanalyzer tool in the virulence factor database (VFDB) using parameters optimized for staphylococcal virulence factors [40,41,42,43,44]. Pairwise genome comparison was carried out with PLASTN using default parameters optimized for highly similar sequences. fPfSau02 sequence was submitted to GenBank with accession no. MK348510.

### 2.4. Production of Phage Cocktail

The three phages used in the phage cocktails were individually produced in liquid medium (mEBHT) or from semi-confluent plates (fRuSau02 and fPfSau02) using standard methods as described elsewhere [35]. The raw lysates were first concentrated three- to four-fold by ultrafiltration with Vivacell 250 ultrafiltration concentrator having 100 kDa PES membrane insert (Sartorius): For mEBHT and fRuSau02, 500 mL of raw lysates was concentrated to 150 mL, and for fPfSau02, 120 mL was concentrated to 30 mL. The concentrated raw lysates were then washed twice with 150 mL SM buffer (100 mM NaCl, 10 mM MgSO_4_, 50 mM Tris-HCl, pH 7.5, 0.01% (*w*/*v*) gelatin). The final volumes of the phages in SM buffer were 40 mL to 90 mL. The phage cocktail HFC-SA1 was prepared by combining the ultrafiltrated lysates at a 1:1:1 PFU ratio. The final theoretical concentrations of the cocktail used for nostril and skin applications were 1 × 10^10^ PFU/mL and 1.4 × 10^8^ PFU/mL for each phage, respectively. To verify that the phages survived spraying, a sample of the phage cocktail meant for skin application was sprayed out from the bottle and titrated in both production strains along with a non-sprayed cocktail.

### 2.5. Selection of the Experimental Animals

The animal experiment was approved by the Project Authorization Board ELLA (project identification code ESAVI/7280/04.10.07/2017). Before the actual trial, a total of 54 weanling pigs of 14–15 kg from a farrow-to-finish farm with 700 sows were sampled from both nostrils. Sampling and subsequent processing of the samples have been described elsewhere [30]. Of all 54 pigs, 38 were confirmed MRSA positive. From these, 20 MRSA-positive pigs were randomly selected and assigned to two groups (*n* = 10 each).

### 2.6. Phage Treatment

Two weeks after the initial testing, the 20 selected weaner pigs were transported to the large animal facility of the University of Helsinki Laboratory Animal Centre. The weight of animals at the time of the transportation was approx. 20 kg. The two groups with ten pigs each were placed in two separate rooms with separate ventilation. In each room, the pigs were kept in two pens of five pigs with constant contact between the pens. Protective clothing for personnel and scientists consisted of disposable overalls with hoods, disposable masks (FFP 2), latex gloves covered with surgical gloves, and boots. Clothing was put on before entering the room and disposed of when leaving. Nothing was taken out or into the rooms without disinfection (Virkon S, LANXESS, Cologne, Germany). Before the start of the experimental phase, the pigs were kept in the rooms for two weeks for assimilation to the new environment. One pig of the control group was lost due to an injury shortly after transport.

The course of the experiment is described in Table 1. To prevent cross-contamination, all procedures were first completed for the control group and then for the test group. For nasal application, 100 µL phage solution or placebo solution (SM buffer) was applied with nasal spray bottles to the nares of test group and control group animals, respectively, on days 28, 30, and 32 of the experiment. The phage dose received by the test group was 1 × 10^9^ PFU for each phage. Larger spray bottles were used to apply six sprays (approx. 1.2 mL each) to the skin: one behind each ear, one to each side of the body, and one on each side of the tail. Test group animals received approximately 1 × 10^9^ PFU of each phage to the skin, control group animals were sprayed with SM buffer.

On day 50 of the experiment, the pigs were sedated with medetomidine (0.1 mg/kg) and ketamine (10 mg/kg) and euthanized with pentobarbital (60 mg/kg). The approx. weight of the pigs at sedation was 62 kg.

### 2.7. Animal Well-Being and the Sample Collection for MRSA, Phage, and Antibody Detection

Body temperature of the pigs was measured rectally for the first three days, after which the decision was made to microchip the pigs with microchips for body temperature monitoring (Lifechip with Bio-Thermo, Destron Fearing, TX, USA) behind the left ear to avoid unnecessary stress. In addition, the overall well-being of the animals was monitored visually daily by the animal caretakers of the facility and kept on record.

To analyze the amount of bacteria and phages in the nostrils and skin of the animals, swab samples were taken during the experiment. The first samples were taken just before the first phage application. Sampling and processing of the samples have been described elsewhere [30]. To determine the formation of anti-phage antibodies during the treatment, blood samples were taken on days 28, 29, and 50. The last samples on day 50 were taken after sedation just before the pigs were euthanized. Blood samples were taken from the vena jugularis into vacuum tubes (BD Vacutainer SST II Advance) and centrifuged 1 h after sampling at 1000 rpm, RT, 10 min (Eppendorf 5804 R Centrifuge). The sera were transferred into new tubes and stored at −20 °C until analyzed.

To study whether phages were able to survive in the litter of the pens, dry samples from the floors of the phage group animals were collected on days 14 (just before the animals were transported to the facility), 28 (before the first phage application), and 50 of the experiment (after the animals were euthanized). The samples were stored at +4 °C until the phage analysis.

### 2.8. Analyzing the Presence of Phages in the Swab Samples

To determine whether skin or nostril samples contained phages, 25 µL of each sample was assayed for plaques on *S. aureus* strains 13KP and 19A2 on soft-agar plates. The resulting plaques were picked and suspended in 100 µL SM buffer. Of this suspension, five µL was used as a template in plaque-PCR for phage identification. Primers used in PCR were fRuSau02_mcp_F (5′-GCCGTCCTGCTCAATCTACA) and fRuSau02_mcp_R (5′-TACGTCTGCTGTTTCAGGCA) for fRuSau02, fPfSau04_mcp_F (5′-AGCGAAAGTTAAAGACACAGGA) and fPfSau04_mcp_R (5′-GCACTGTCTAATGTACGTTGCT) for fPfSau02, and EBHT-tp-for (5′-AGCGTGATTTCGGGTCGCTA) and EBHT-tp-rev (5′-AGTGGCATGACGCACAAGG) for mEBHT. PCR was performed with DreamTaq DNA polymerase (Thermo Fisher Scientific) using conditions recommended by the manufacturer.

To analyze the presence of phages in the litter samples collected from the floor of the pens, 0.5–1.5 g of solid sample material was suspended in a 17-x volume of SM buffer. After overnight incubation at +4 °C, the samples were centrifuged, and the supernatants were collected and sterile-filtered. 1.5 mL of the supernatants were used for phage enrichment with a mixture of strains 13KP and 19A2, after which phages were assayed in both strains as above.

### 2.9. Analyzing the Bacteria in the Swab Samples

Nose and skin swabs were processed both by direct plating and enrichment as described in [30]. Species confirmation, extraction of genomic DNA, and confirmation of MRSA by *mec* PCR were performed as described elsewhere [30].

### 2.10. Detection of Phage Antibodies in Pig Serum Samples

Antibody detection from pig serum samples collected on days 28, 29, and 50 of the experiment, was carried out with ELISA on 96-well plates (High Binding Isoplate-96 HB Black Frame) with phages mEBHT and fRu-Sau02. The ELISA assay was first optimized with phage фYeO3-12, for which rabbit antiserum was available [32]. To prepare the ELISA plates, both phages were first produced as 500 mL lysates in LB and purified with PEG -precipitation [35] and twice with glycerol step gradient ultracentrifugation [35].

To set up and optimize an ELISA assay for antibody detection, a 96-well plate (Isoplate-96 F HB, PerkinElmer, Waltham, MA, USA) was coated with фYeO3-12 phage with two different phage concentrations, 5 × 10^9^ PFU and 1 × 10^10^ PFU in 200 µL, respectively. Each condition was tested as duplicate. The wells were blocked with 100 µL MAXblockTM Blocking medium (Active Motif, Carlsbad, CA, USA) for 3 h at RT. Both phage concentrations were tested with six different primary antibody dilutions (1:100, 1:500, 1:1000, 1:2000, 1:5000, and 1:10,000) in PBS. Of each dilution, 75 µL was used for the assay. For detection, 75 µL of Alexa Fluor 488-labeled secondary antibody (Alexa Fluor 488-conjugated AffiniPure Goat Anti-Rabbit IgG (H + L) (Jackson ImmunoResearch Laboratories, West Grove, PA, USA), diluted 1:200 in PBS, was used. The plate was incubated for 90 min at RT protected from light. Finally, fluorescence was measured with Hidex Plate CHAMELEON Multilabel Detection Platform (Hidex, Turku, Finland) using A488 settings.

The method described above was then applied for the antibody detection from pig serum samples. To this end, each well was coated with 5 × 10^9^ PFU one of the tested phages in PBS, and serum samples diluted to 1:10 were used. Each sample was tested in triplicates. Alexa Fluor 488-conjugated AffiniPure Goat Anti-Swine IgG (H + L) secondary antibody (Jackson ImmunoResearch Laboratories, West Grove, PA, USA) was used for detection.

## 3. Results

### 3.1. Selection of the Phages Used in Phage Cocktail HFC-SA1

We aimed to produce a phage cocktail that would have as wide a host range against Finnish LA-MRSA strains as possible. To this end, the host range of *S. aureus*-specific phages was analyzed with 92 LA-MRSA strains isolated one year earlier from the same pig farm [30]. The results showed that phages fPfSau02 and fPfSau03 had identical host ranges, both infecting 91 out of 92 tested strains (Supplementary Materials Table S1). Phage fPfSau04 infected 89 strains, four out of which very weakly. fRuSau02 infected well 49 strains and weakly six strains, and phage mEBHT infected well 80 strains and 11 strains weakly.

Preliminary sequence analysis of fPfSau02, fPfSau03, and fPfSau04 showed that they all had 45.1 kb genomes. Phages fPfSau02 and fPfSau03 were 100% identical to each other and were thus found to be the same phage, and fPfSau04 was 99.99% identical to them. As fPfSau02 (fPfSau03) had a wider host range than fPfSau04, we decided to continue to work with only fPfSau02. The phage belongs to the *Triavirus* genus in the *Siphoviridae* family, and it has a clearly identifiable site-specific tyrosine-integrase. Therefore, it is a temperate phage and would not be applicable to a real phage therapy treatment. However, as this work only concerned experimental animals in a closed environment, fPfSau02 had no identifiable virulence factors in its genome, and it had a very wide host range among the MRSA strains, we decided to include it in the phage cocktail HFC-SA1. The other two phages in the cocktail, fRuSau02 and mEBHT, were structurally very different from each other and infected most tested LA-MRSA strains. Therefore, we considered their inclusion in the cocktail justified.

The theoretical titers of phage cocktails meant for nasal and skin applications were 1 × 10^10^ PFU/mL and 1.4 × 10^8^ PFU/mL for each phage, respectively. The titration results after mixing the cocktail in 19A2 strain (showing mEBHT and fPfSau02) were 1 × 10^10^ PFU/mL and 2 × 10^8^ PFU/mL in nasal and skin bottles, respectively. The corresponding titers in strain 13KP (fRuSau02 phage) were 2 × 10^9^ PFU/mL and 1 × 10^7^ PFU/mL. It thus seemed that titers in 19A2 were very close to the theoretical values but phage fRuSau02 was inhibited by five- to tenfold. To verify that the phages survived spraying from the skin bottles, aliquots of the cocktail were titrated before and after spraying in both production strains. The titer drops in strains 19A2 and 13KP were 6.7% and 27%, indicating that no major loss in titer occurred.

### 3.2. The General Well-Being of the Animals during the Experiment

The animal well-being was monitored throughout the experiment, and nineteen out of twenty pigs showed no signs of disease, change in the body temperature, or other unexpected side effects. Unfortunately, one animal belonging to the control group suffered from a leg injury during the transportation, and it had to be euthanized before the beginning of the actual phage experiment.

### 3.3. Phages Were Recovered from the Animals in the Experimental Group on the Next Days following Phage Applications

**Figure 1:**
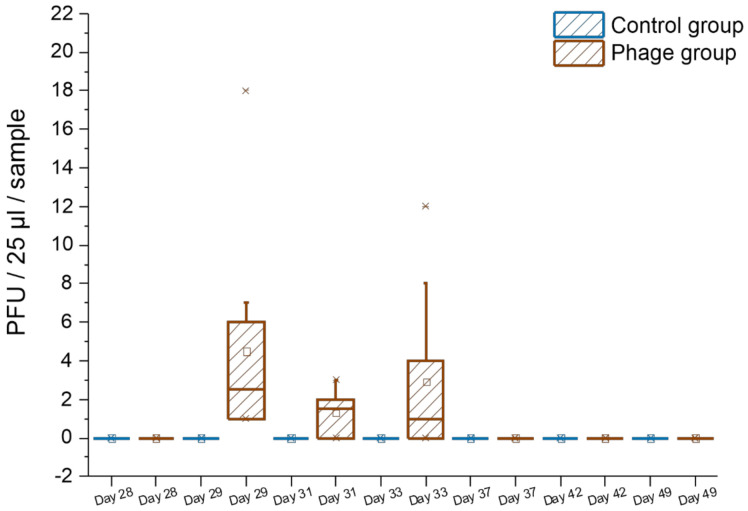
Number of phage PFUs in nasal and skin swab samples. For each nasal and swab sample, 25 µL was analyzed for the presence of phages in *S. aureus* strains 13KP and 19A2 by the double-agar method. Plaques were only observed in strain 19A2. Square, horizontal line, box boundaries, vertical lines, and crosses indicate mean, median, upper and lower quartiles, upper and lower 95% values, and maximal and minimal values, respectively.

**Figure 2:**
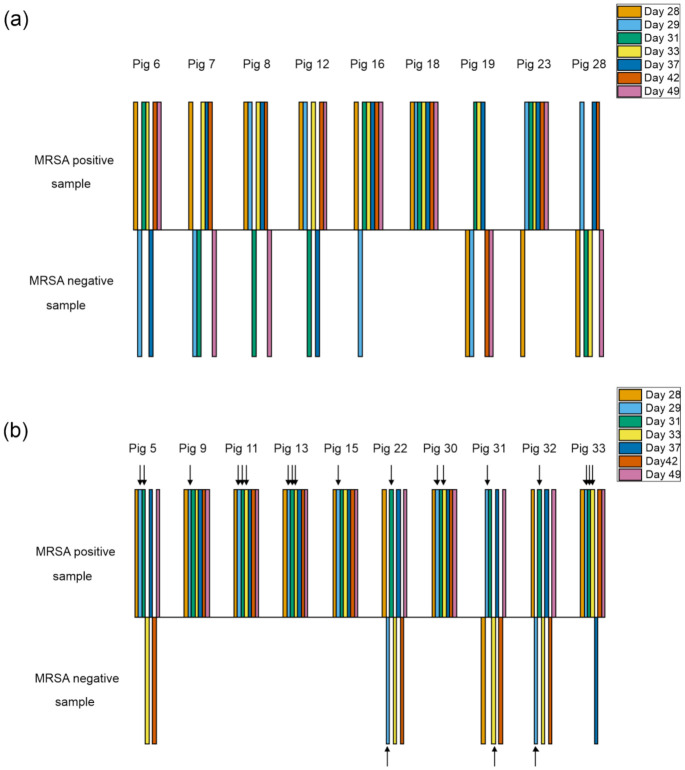
The distribution of MRSA and phage in the nasal and swab samples during the experiment. The results from the control group (**a**) and phage group (**b**) are shown. Bars above and below the line indicate MRSA positive and negative samples, respectively. An animal was considered positive if MRSA was detected in either nasal swab sample at a given time point. The black arrows indicate positive phage findings in the same samples.

In order to study if the phages used in the cocktail HFC-SA1 survived on pig skin and nostrils, 25-µL aliquots of the swab samples collected during the experiment (Table 1) were tested on *S. aureus* strain 13Kp for the presence of fRuSau02 and on strain 19A2 for the presence of mEBHT and fPfSau02. The animals belonging to the control group had no plaques at any time point, whereas all the phage group animals had phages in at least one nasal or skin swab sample (Figure 1 and Figure 2). An animal having phages in at least one of the samples, either skin swab or nasal, was considered phage-positive. Phages were only observed in samples taken on days 29, 31, and 33, i.e., the days immediately following the phage applications. The phage numbers in the samples were very low, only 1–13 PFU/25 µL-sample (Figure 1). The proportion of phage-positive samples and the titer of phages was similar in nasal and skin swab samples (not shown). We could only observe phage plaques on strain 19A2, which was sensitive to phages mEBHT and fPfSau02 but not to fRuSau02. We did not detect phage plaques in the litter samples collected from the floor of the pens on either strain 13Kp or 19A2.

To further analyze which out of the three phages dominated in the experimental setting, all 54 plaques obtained from nasal and skin swab samples were picked and the identity of the phages was tested with PCR. As expected, we did not detect fRuSau02 in any of the plaque samples. Interestingly, all the analyzed plaques contained mEBHT, indicating that mEBHT clearly dominated over the other two phages. Phage fPfSau02 was detected in only one sample, which was positive for both fPfSau02 and mEBHT (not shown).

### 3.4. Phage Treatment Did Not Eradicate MRSA from Healthy Carrier Pigs

As shown in Figure 2, the phage treatment did not reduce the number of MRSA-positive animals. Eight out of nine animals (89%) in the control group and five out of ten (50%) animals in the phage group were MRSA-negative at least at one time point during the experiment. Not a single animal was found MRSA-negative for the whole follow-up time. On the contrary, one animal in the control group and five animals in the phage group gave an MRSA-positive sample at each time point.

Our recent study showed that typical bacterial concentrations in the directly plated skin swab samples in the healthy MRSA carrier animals were 10–100 CFU/swab samples [30]. Considering that 5 cm × 5 cm skin region was swabbed for each sample, this would correspond to less than 10 CFU/cm^2^. The concentrations in nasal samples were at a similar level to in the swab samples. The bacterial load in these healthy carrier animals was thus very low. In this work, we could not observe any difference in the MRSA concentrations between the MRSA -positive animals in the control and phage groups, and all detected bacterial loads were similar to the previous work (not shown).

In the animals belonging to the phage treatment group, phages were observed in both MRSA-negative and -positive samples (Figure 2b). Furthermore, a phage-positive finding did not predict an MRSA-negative sample in the next time point.

### 3.5. MRSA Strains Collected during the Phage Treatment Were Still Sensitive to the Phages Used in the Cocktail

To analyze whether the MRSA strains colonizing the pigs became phage-resistant during the experiment, the sensitivities of the strains isolated from the swab samples to phages fRuSau02, mEBHT, and fPfSau02 individually and to the cocktail HFC-SA1 were determined (Supplementary Materials Table S2). Altogether 276 strains were tested, 110 of which were isolated from the control group and 166 from the phage group animals. As shown in Supplementary Materials Table S2, all the strains were sensitive to the cocktail HFC-SA1 and at least one of the phages, even though for some strains the phage infection was weak. Thus, phage resistance was not detected in the MRSA strains during the phage treatment.

The infectivities of the individual phages used in the cocktail varied to some extent. Phage mEBHT infected clearly 79 of the 110 strains isolated from the control group (72%) and 108 of the 166 strains isolated from the phage group (65%). In addition, two strains (1.2%) isolated from the phage group were infected weakly. Phage fRuSau02 infected all the strains analyzed, but the infection was weak in 93 (85%) and 103 (62%) strains isolated from the control and phage groups, respectively. Phage fPfSau02 infected all the strains isolated from the control group and all but one strain from the phage group. As opposed to fRuSau02, the infection of fPfSau02 was clear in 99 (90%) and 147 (89%) of the strains isolated from the control and phage groups, respectively. Its infection was weak in only 11 (10%) and 19 (11%) of the control and phage group strains, respectively.

When comparing the infection profile of the cocktail HFC-SA1 to the infection profiles of the individual phages, an interesting observation became evident: All the strains that were only weakly infected by the cocktail were resistant to mEBHT and weakly infected by fRuSau02. The infection profile of fPfSau02 in these strains did not affect the infectivity of the cocktail. This finding indicates that a clear infection by fPfSau02 was not enough for clear infection by the cocktail, and the other two phages may have interfered with its infection. The findings that the fRuSau02 had poor overall infectivity in these LA-MRSA strains and that the fPfSau02 infection in the cocktail was inhibited correlate with the results that all phage samples collected during the experiment were positive for mEBHT. mEBHT was thus the predominant phage in this experiment.

### 3.6. Pigs Did Not Produce Antibodies against the Phages Used in the Cocktail

In order to find out whether the pigs developed phage-specific antibodies during the experiment, serum samples were collected from the animals belonging to both the control and phage groups on days 28, 29, and 50 (Table 1). Antibodies against phages mEBHT and fRuSau02 were assayed by an in-house ELISA test.

**Figure 3:**
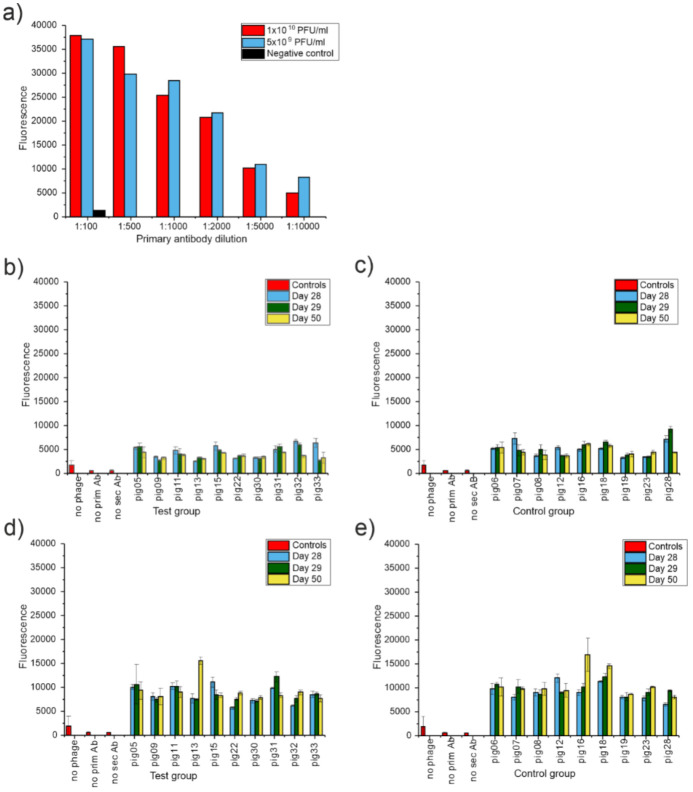
An in-house ELISA assay for the detection of phage-specific antibodies in serum samples. (**a**) The detection of ΦYeO3-12 specific antibodies in rabbit antiserum in wells coated with 5 × 10^9^ PFU and 1 × 10^10^ PFU of purified phage ΦYeO3-12. The result shown is a mean of two measurements. (**b**) The detection of mEBHT antibodies in phage group animals. (**c**) The detection of mEBHT antibodies in control group animals. (**d**) The detection of fRuSau02 antibodies in phage group animals. (**e**) The detection of fRuSau02 antibodies in control group animals. Negative controls included the assay performed with no phage used for coating and no primary or secondary antibody used for detection. In (**b**–**e**), the results shown are means of three measurements and the error bars indicate standard deviation.

The results demonstrated that the pigs did not produce detectable levels of antibodies against either phage mEBHT or fRuSau02 during the experiment (Figure 3). There were no differences in the signal levels between the control and phage group pigs at any time point. In addition, there were no increases in the signals between the samples taken before the first phage application and at day 50 of the experiment (i.e., 4 weeks after the first phage application) in the phage group.

## 4. Discussion

LA-MRSA, especially the strains belonging to CC398, can colonize several species of production animals and may spread in the human population by being transmitted, e.g., by animal caretakers and veterinarians. These strains may thus pose health risks for immunocompromised or otherwise vulnerable people [6,7,8,9]. In this work, our aim was to evaluate the efficacy of phage therapy in the eradication of low-level nasal and/or skin MRSA carriage in pigs.

In order to study the effect of phage applications on the MRSA colonization levels, nineteen pigs with earlier confirmed MRSA carriage [30] were divided into control and phage groups in an isolated animal facility. The animals were treated three times on every second day by spraying either buffer or a cocktail of *S. aureus*-specific phages to the nares and skin. The phage cocktail consisted of three phages belonging to different morphotypes. The bacterial and phage counts in the nasal and skin swab samples were followed for three weeks, and the phage sensitivity of the MRSA strains was tested. In addition, blood samples taken before and after the test period were analyzed for the presence of phage-specific antibodies. The animals did not show any signs of illness in either group during the experiment follow-up period, indicating that the phage treatment did not cause any side effects.

Phages used in the cocktail were found to survive in the animals for one day after the application, but no phages were observed at later time points. We detected phages in both nasal and skin samples in every animal belonging to the phage group but not in the control group animals. Of the three phages used in the cocktail, the small podovirus mEBHT clearly dominated over fRuSau02 and fPfSau02. We did not observe fRuSau02 in any of the follow-up samples, most probably reflecting the observation that this phage infects human-originating *S. aureus* isolates much more efficiently [31] than the pig isolates analyzed in this work. This was a rather unexpected finding, as K-like viruses were earlier found to have a wide host range even among LA-MRSA strains [45]. In addition, the finding that the infectivity of fRuSau02 in the cocktail was decreased to some extent even when titrated in strain 13KP, which is resistant to the other two phages, may explain why we did not detect fRuSau02 in the follow-up samples.

The poor survival of fPfSau02 (only one positive sample) was slightly surprising, as it infected the strains analyzed in this work very efficiently when tested separately. However, it seems that a clear infection by fPfSau02 separately resulted only in faint infection by the cocktail in strains that were not infected efficiently by mEBHT or fRuSau02, indicating that the other two phages inhibited fPfSau02 in the cocktail. The mechanism of this inhibition is not known, but it may result from either the competition of receptors, differences in adsorption efficiency, or superinfection exclusion.

As fPfSau02 is a temperate phage, there was naturally a possibility that lysogens might have been formed in the strains isolated from the phage group pigs. However, the phage was able to infect all but one out of the 166 strains isolated from the phage group, which strongly argues against the formation of lysogens. Interestingly, further analysis of the genomic sequence of strain 19A2 [30], the production host of fPfSau02, revealed that this strain harbors a prophage that is 95% identical to fPfSau02 (not shown). The genomic regions that differ most between these two phages include regulatory genes that participate in the maintenance of the lysogenic state. For example, the fPfSau02 locus PFS2_028, coding for a putative phage repressor protein C, is completely missing from the putative prophage, which harbors a shorter XRE-family-like protein instead. Differences in this region may explain why lytic fPfSau02 infection in this strain is not blocked by superinfection immunity. We randomly selected four more fPfSau02–sensitive strains isolated from the phage group pigs (F13A1, F30A2MH, F15A3, and F11A7MH, Supplementary Materials Table S2), and checked the presence of an fPfSau02–like prophage by colony-PCR with fPfSau02–specific primers. All four strains produced positive signals in this PCR (not shown), indicating that fPfSau02–like prophage (or remnants of it) may be widespread among these strains. As the integrases of fPfSau02 and this putative prophage are 100% identical (not shown), they probably utilize the same attachment site. The presence of this prophage may thus prevent fPfSau02 from forming lysogens in these strains. However, this hypothesis should be experimentally confirmed before any definite conclusions can be drawn.

The MRSA levels stayed similarly low in both phage and control groups for the whole experiment. It thus clearly seems that phage treatment did not decrease the bacterial counts, and phages were not able to reproduce themselves in the nares and skin of the pigs. This finding is similar to the earlier result by Verstappen et al., who did not observe a reduction in the porcine nasal MRSA colonization after phage treatment [28]. In our work, the host coverage of the phage cocktail was extremely wide, and all MRSA strains collected from the animals during the follow-up period were still sensitive to at least one of the phages. Furthermore, we did not detect phage-specific antibodies in the serum samples. These indicate that phage resistance or neutralizing antibodies were not reasons for the poor efficacy of the treatment. In earlier studies, the replication threshold of phages has been estimated to be around 10^4^ CFU/mL (i.e., the phage can only replicate if the concentration of susceptible bacteria is higher than 10^4^ CFU/mL) [46,47,48]. In this and our earlier/partially parallel work [30], the MRSA concentrations were found to be surprisingly low, only 10–100 CFU/swab or nasal sample. Even though the bacterial quantities in at least the nasal samples suffered from a certain degree of unreliability due to challenges in sampling, the concentrations were clearly below the threshold of 10^4^ CFU/mL. In the conditions of such a low bacterial concentration, phages cannot find their host bacteria and do not replicate. They are thus eliminated from the skin and nares of the animals without being able to establish themselves.

The low bacterial counts in our study may not allow conclusions if a higher MRSA colonization level could be significantly reduced by phage therapy, e.g., before slaughter. However, it can be deduced that phage treatment may not be an efficient way to eradicate bacteria from healthy carriers, at least in situations where the bacterial concentrations are very low, and bacteria do not stimulate an immune response against them.

## Figures and Tables

**Table 1:** The course of the phage therapy trial. Numbers indicate the day of the experiment.

Operation	0	14	28	29	30	31	32	33	37	42	49	50
Initial Testing	×											
Transfer of Selected Pigs		×										
Start of the PT Experiment *			×									
Phage or Placebo Treatment			×		×		×					
Skin and Nose Swabs			×	×		×		×	×	×	×	
Blood Sample			×	×								×
Environmental Sample		×	×									×
Rectal Temperature			×	×	×							
Microchipping the Pigs						×						
Body Temperature						×	×	×	×	×	×	
Sedation												×
Euthanasia												×

* PT; Phage therapy.

## Data Availability

Data is contained within the article and supplementary material. The genomic sequences of phages used in the work have been submitted to GenBank with accession numbers MK348510 (fPfSau02), MT926124 (mEBHT), and MF398190 (fRuSau02).

## Supplementary Materials

The following are available online at https://www.mdpi.com/article/10.3390/v13101888/s1, Table S1: The infectivity of *S. aureus*–specific phages in LA-MRSA strains collected one year prior to the animal experiment. Table S2: The sensitivity of the MRSA strains isolated during the phage therapy experiment to the cocktail HFC-SA1 and the individual phages used in it.
